# Chronic Vaginitis—Treatment

**Published:** 1874-08

**Authors:** George T. Harrison


*VIRGINIA MEDICAL MONTHLY*- *July*, 1874.

Chronic Vaginitis.—*By George T. Harrison, M.D.*— * * * In treating chronic vaginitis, we must have regard, in the first instance, to its causes. If it is combined with chronic endometritis, changes in the position of the uterus, etc., or if due to chlorosis, treatment must be directed to these affections primarily. Local treatment, however, can rarely be dispensed with; and when we have to deal with obstinate cases, as especially when the touch reveals thickening of the walls of the vagina, and hemispherical elevations thickly strewn over its surface, it is idle to waste time with weak solutions of zinc, alum, nitrate of silver, with tannin suppositories, etc. We must proceed at once to the use of the stronger caustics—either chromic acid or nitrate of silver in stick. If chromic acid is used (I generally use it diluted with an equal quantity of water), the walls of the vagina are exposed to view by the speculum, and a thorough application made to the diseased surface. A piece of cotton saturated with glycerine is then introduced into the vagina, and a piece of string attached to the cotton to enable the patient to draw it out the next morning. Copious injections of flaxseed tea should be ordered twice or thrice daily, used at a temperature of 98°F. Hot water injections should also be enjoined twice daily during the continuance of the treatment. The nitrate of silver causes a good deal of pain for one, two or three hours; when excessive, a solution of common salt should be thrown into the vagina. I use the nitrate of silver in stick in preference to strong solutions of the crystalized form. A glycerine dressing should be introduced after its use, as after the chromic acid.

After the immediate effects of the cauterization have disappeared—and in the case of chromic acid it may require a week—one of Sims' dilators, such an one as he uses after his operation for vaginismus, is introduced, and the patient directed to wear it continuously. It should be dipped in warm water and then in glycerine before being introduced. In obstinate cases, simple cauterization will disappoint our expectations; but if succeeded by the use of the glass, the worst cases will yield sooner or later. Obviously, I leave out of view such cases where the disease depends upon constitutional affections, which are incurable.

The favorable action of the dilator is three-fold. In the first place, by keeping the inflamed walls of the vagina apart, rest is secured; secondly, with the distension of the vaginal walls, compression is exercised upon the blood vessels distributed to them, and the quantity of blood contained in them diminished, or, in other words, the hyperæmia is moderated; and thirdly, the pressure brought to bear upon the hyperplastic tissue causes its atrophy, or, to speak after the manner of the pathologist, induces regressive changes in it. The size of the glass dilator must be adapted to the requirements of the individual case. It is possible to put in such a tight-fitting vaginal plug as to cause sloughing, and it should be secured *in situ* by a T bandage. After the use of the glass dilator for a week or more, an improvement of a marked character will be at once apparent. If the cauterization has to be repeated, it should be followed by flaxseed tea injections and glycerine dressings for several days before again using the dilator. The length of time during which the dilator should be worn will depend upon the gravity of the affection. A few weeks may suffice, or it may require months to affect our purpose.

